# Impact of post-COVID-19 olfactory disorders on quality of life, hedonic experiences and psychiatric dimensions in general population

**DOI:** 10.1186/s12888-024-05538-0

**Published:** 2024-02-08

**Authors:** Louise-Emilie Dumas, Clair Vandersteen, Victoria Metelkina-Fernandez, Auriane Gros, Philippe Auby, Florence Askenazy-Gittard

**Affiliations:** 1 Service Universitaire de Psychiatrie de l’Enfant et de l’Adolescent (SUPEA), Hôpitaux Pédiatriques de Nice, Centre Hospitalier Universitaire-Lenval, Nice, France; 2 CoBTeK-Lab, Université Côte d’Azur, Nice, France; 3 Institut Universitaire de la Face et du Cou (IUFC), ENT Department, Centre Hospitalier Universitaire, Nice, France; 4 Service de Psychiatrie, Centre Hospitalier Universitaire, Nice, France; 5 Département d’Orthophonie (DON), Université Côte d’Azur, Nice, France

**Keywords:** Olfactory disorders, Odor perception, Covid-19, Psychiatric, Psychopathology, Emotion, Cognition, Hedonic experiences (8 words)

## Abstract

**Background and objective:**

Olfactory disorders in COVID-19 impact quality of life and may lead to psychological impairments. Prevalence ranges from 8 to 85%, persisting in about 30% of cases. This study aimed to evaluate the 6-month post-COVID-19 impact on quality of life, hedonic experiences, anxiety and depression due to olfactory disorders. Additionally, it sought to compare psychophysical tests and self-perceived olfactory evaluations.

**Methods:**

A prospective, longitudinal study was conducted over baseline (T0) and 6 months (T1) on individuals with persistent olfactory disorders post-COVID-19 for more than 6 weeks. Psychophysical tests employed the Sniffin’ Sticks Test® (TDI score), and self-perceived olfactory evaluation used a Visual Analogue Scale. Quality of life was assessed with an Olfactive Disorder Questionnaire and the French version of the Quality of Life and Diet Questionnaire. Hedonic experiences were gauged using the Snaith-Hamilton Pleasure Scale, while anxiety and depression dimensions were measured by The State-Trait Anxiety Inventory, The Post Traumatic Stress Checklist Scale, and Hamilton Rating Scale for Depression. Participants were classified into the “normosmic group” (NG) and the “olfactory disorders group” (ODG) at T0 and T1 based on the TDI score.

**Results:**

Were included 56 participants (58.93% women, 41.07% men) with a mean age of 39.04 years and a mean duration of post-COVID-19 olfactory disorders of 5.32 months. At T1, ODG had a significantly lower quality of life and hedonic experiences than NG. No significant differences in anxiety and depression dimensions were observed between groups. At T0, psychophysical tests and self-perceived olfactory evaluations were significantly correlated with quality of life and hedonic experiences in both groups. At T1, self-perceived olfactory evaluation in NG correlated significantly with quality of life, hedonic experiences, anxiety and depression dimensions, whereas ODG only correlated with hedonic experiences.

**Conclusion:**

Individuals with persistent post-COVID-19 olfactory disorders after six months demonstrated compromised quality of life and hedonic experiences. Self-perceived olfactory evaluation played a more significant role in influencing quality of life and the dimension of anxiety and depression than the psychophysical presence of olfactory disorders. These findings emphasize the importance of considering patients’ perceptions to comprehensively assess the impact of olfactory disorders on their well-being.

**Trial registration:**

ClinicalTrials.gov number (ID: NCT04799977).

## Introduction

The SARS-CoV-2 coronavirus, responsible for COVID-19, has had a global impact on the population, resulting in various acute and chronic symptoms [1]. Among these symptoms, olfactory disorders have been reported, with a highly variable prevalence ranging from 8 to 85%. This variability can be attributed to factors such as age, race, gender, vaccination status, smoking habits, genetic factors, time since the onset of infection, comorbidities, and specific COVID-19 variants [2–4]. Moein et al. discovered that, while 61% of the 82 study participants had recovered their sense of smell within 7–8 weeks after the onset of COVID-19, the remaining 39% continued to experience olfactory disorders [5]. Additional data from the literature also reveals that olfactory disturbances persist in 25–30% of COVID-19 cases [6] and remain stable for 1–6 months [7]. In a study involving 268 COVID-19 patients, 21.9% reported that their olfactory function had not returned to normal even one year after the initial diagnosis [8].

Olfactory disorders (OD), defined as the reduced or distorted ability to smell during sniffing (orthonasal olfaction) or eating (retronasal olfaction) [9], are associated with various adverse effects on the quality of life. Regardless of their underlying cause, olfactory disorders result in diminished food enjoyment, compromised safety and hazard awareness, challenges in maintaining personal hygiene, occupational limitations, and social isolation. Additionally, they can lead to psychological issues such as insomnia, anxiety, and depression, as well as cognitive impairments affecting frontoparietal cognitive functions and increasing the risk of neurodegenerative diseases [10–13].

Research has consistently shown higher levels of depression and anxiety among individuals with olfactory impairment, often resulting in social withdrawal [10, 14]. Furthermore, studies have established a correlation between the severity of olfactory disorders and the intensity of depressive symptoms [15]. The literature also reports that olfactory disorders during the acute phase of COVID-19 infection are linked to cognitive deficits and psychiatric disturbances [16].

In our study, we initiated the assessment of patients during the COVID-19 pandemic in 2020. Early on, we formulated a hypothesis that individuals experiencing post-COVID-19 olfactory complaints might be linked to a decline in their quality of life and exhibit psychiatric symptoms. Our observations align with the experiences shared by patients and have been subsequently described in the literature. Patients have reported experiencing a general sense of mental fatigue, a loss of enjoyment in the taste of food and in social interactions during mealtimes, a lack of comforting scents, and, in some cases, aversion to unpleasant and distorted odors [6, 17–21]. Based on our findings and the existing literature, we propose that olfactory disorders may contribute to the development of psychiatric symptoms.

The main objective of this study is to evaluate the impact at 6 months of the post COVID-19 olfactory disorders on several criteria: quality of life, hedonic experiences and dimensions of anxiety and depression dimensions in the general population. The second objective is to compare psychophysical tests and self-perceived olfactory evaluation of olfactory disorders using these same criteria.

## Materials and methods

### Study design/setting

This prospective longitudinal open-label cohort study, spanning a duration of six months, was conducted by a scientific consortium consisting of a multidisciplinary team of researchers and healthcare professionals, including psychiatrists, ENT specialists, infectious disease specialists, and speech therapists, among others. The study took place at the Nice University Hospital, running from October 2020 to October 2021.

### Participants

The study included adult participants who had previously contracted COVID-19 and were experiencing persistent olfactory disorders for a duration exceeding six weeks. Most of the patients either referred themselves or were referred by general practitioners. The initial confirmation of COVID-19 infection was obtained through RT-PCR testing, followed by secondary confirmation through serology. Patients were included in the study when they reported ongoing olfactory disorders six weeks after the resolution of COVID-19 symptoms. Exclusion criteria for the study were: pre-existing hyposmia or anosmia prior to contracting COVID-19, a history of sinus or cranial neoplasia, prior sinus or cranial radiotherapy, a neurodegenerative disease diagnosis, or a history of post-viral hyposmia or anosmia that had subsequently recovered.

### Assessment

#### Olfactory disorder assessment

Studies concerning post-COVID-19 olfactory disorders list the different methods of measuring olfactory disorders [22, 23]. In this study, the aim is to compare the methods of psychophysical tests and self-perceived olfactory evaluation. The psychophysical tests of olfactory dysfunctions of the study participants were assessed using the “Sniffin’ Sticks Test®” [24]. This psychophysical olfactory tests determines: the olfactory threshold level (T), olfactory discrimination abilities (D) and olfactory identification (I) in the form of an overall TDI score [25, 26]. The diagnosis of olfactory disorders is retained at a score ≤ 30.5 and thus includes anosmic and hyposmic disorders.

A visual analogue scale (VAS) was used to assess self-perceived olfactory evaluation of olfactory disorders. Patients indicated a score measuring the intensity of olfactory disturbances since COVID-19 infection: 0% when they smelled nothing and 100% when they smelled like before COVID-19. The literature reports that the VAS in post-COVID-19 olfactory disorders is a reliable diagnostic tool, simple and quick to apply [27, 28] as well as strong statistical power [29].

### Quality of life assessment

The Olfactive Disorder Questionnaire (QOD) is a validated quality of life questionnaire centered on the consequences of a loss of smell and taste, in particular in a post-viral situation, such as the pleasure of sharing a meal, of creating social interactions or of forming intimate ties with others [30]. A short version (QODC) was proposed in 7 most relevant questions concerning the social aspect, diet, anxiety as well as boredom following olfactory loss [31]. It was validated in French [32, 33]. The responses are rated from 0 to 3 according to their importance for a total score ranging from 0 to 21 (21 reflecting no alteration in the quality of life related to olfaction).

The French quality of life and diet questionnaire (QV-AF) is validated in French and enables to assess the quality of life in adults, in particular the relationship to food [34]. The QV-AF questionnaire contains four subscales measuring quality of life in relation to food: pleasure, relationship, psychology, physical condition. The maximum total score is 100 per subscale and the maximum score on the questionnaire is 400, corresponding to the best possible quality of life.

### Hedonic experiences

The Snaith-Hamilton Pleasure Scale (Shaps) is the most commonly used in the measurement of anhedonia [35]. This is a 14-item self-administered questionnaire designed to assess the patient’s hedonic capacity in different circumstances of daily life. It is validated and translated into French [36] and covers four domains of hedonic experience: interests/hobbies, social interaction, sensory experience, and food/drink. A score of 3 or more indicates a significant reduction in hedonic capacity and appears to have discriminant value between controls and clinically depressed patients.

#### Psychiatric assessments

The State-Trait Anxiety Inventory (STAI) is a psychological inventory consisting of 40 self-report items on a 4-point Likert scale [37], translated and validated in French [38]. The STAI measures two types of anxiety: state anxiety and trait anxiety. Higher scores are positively correlated with higher levels of anxiety. Each score can therefore vary from 20 to 80 with an anxiety standard: “very high” when the threshold is > 65; “high” between 56 and 65; “average” between 46 and 55; “weak” between 36 and 45 and “very low” for a score < or = 35.

The Post Traumatic Stress Checklist Scale (PCL-5) is a self-report measuring the 20 symptoms of post-traumatic stress disorder (PTSD) from the DSM-5 [39, 40]. The objectives of the PCL-5 are multiple: to screen people with PTSD or to monitor the evolution of symptoms during and after treatment. The PCL-5 scale is validated and translated into French [41, 42]. The questionnaire includes 20 items on a 5-point Likert scale and a threshold of 33 is proposed for screening for post-traumatic stress disorder (PTSD).

Hamilton Rating Scale for Depression (HDRS) [43], translated into French [44], is used to assess the severity and evolution of a patient’s depressive state during a structured interview. This is a hetero-questionnaire consisting of 17 items completed by the examiner during the interview. The higher the score, the more severe the depression: from 10 to 13: depressive symptoms are mild; from 14 to 17: depressive symptoms are mild to moderate; above 18: depressive symptoms are moderate to severe.

### Study procedures

Patients meeting the inclusion criteria underwent two separate and consecutive consultations with an ENT specialist and a psychiatrist. During the ENT specialist’s consultation at T0, the assessment of olfactory disorder was conducted using the “Sniffin’ Sticks Test®” (TDI), the Visual Analog Scale (VAS), and the evaluation of quality of life using the QODC questionnaire. In addition to these assessments, patients received information about their health status and were provided with recommendations for olfactory disorder rehabilitation.

The psychiatrist’s consultation at T0 focused on evaluating quality of life using the QV-AF questionnaire and assessing psychiatric dimensions using the STAI (State-Trait Anxiety Inventory), PCL-5 (Posttraumatic Stress Disorder Checklist-5), SHAPS (Snaith-Hamilton Pleasure Scale), and HDRS (Hamilton Depression Rating Scale). During this consultation, psychiatric disorders could be diagnosed, initially through the results of the scales and subsequently through clinical judgment. Patients diagnosed with psychiatric disorders were informed and referred to a therapist for treatment.

Six months later at T1, all participants in the study were contacted for a follow-up assessment, which was conducted by the ENT specialist and psychiatrist using the same assessment methods as in the initial T0 evaluation. At both T0 and T1, participants were categorized into the “normosmic” group (NG) if their TDI score > 30.5, or into the “olfactory disorder” group (ODG) if their TDI score ≤ 30.5.

### Statistical analysis

The ENT specialist and psychiatrist directly enter the data into the database during the evaluations. The hypothesis is that post-COVID-19 olfactory disorders impair quality of life and hedonic experiences and lead to symptoms of anxiety and depression dimensions. Statistical analyses were performed using R software.

Differences in NG and ODG were assessed at T0 and T1 in independent samples. Given that the data did not follow a normal distribution, Wilcoxon’s test was used to compare the means of the NG and ODG.We retained a significant difference between the two populations with *p* < 0.05. Correlation test (rho) (with spearman method) measured correlation between psychophysical tests (TDI) and self-perceived olfactory evaluation (VAS) of olfactory disorder and quality of life, hedonic experiences, anxiety and depression dimensions in each group (NG and ODG).

## Results

**Fig. 1:**
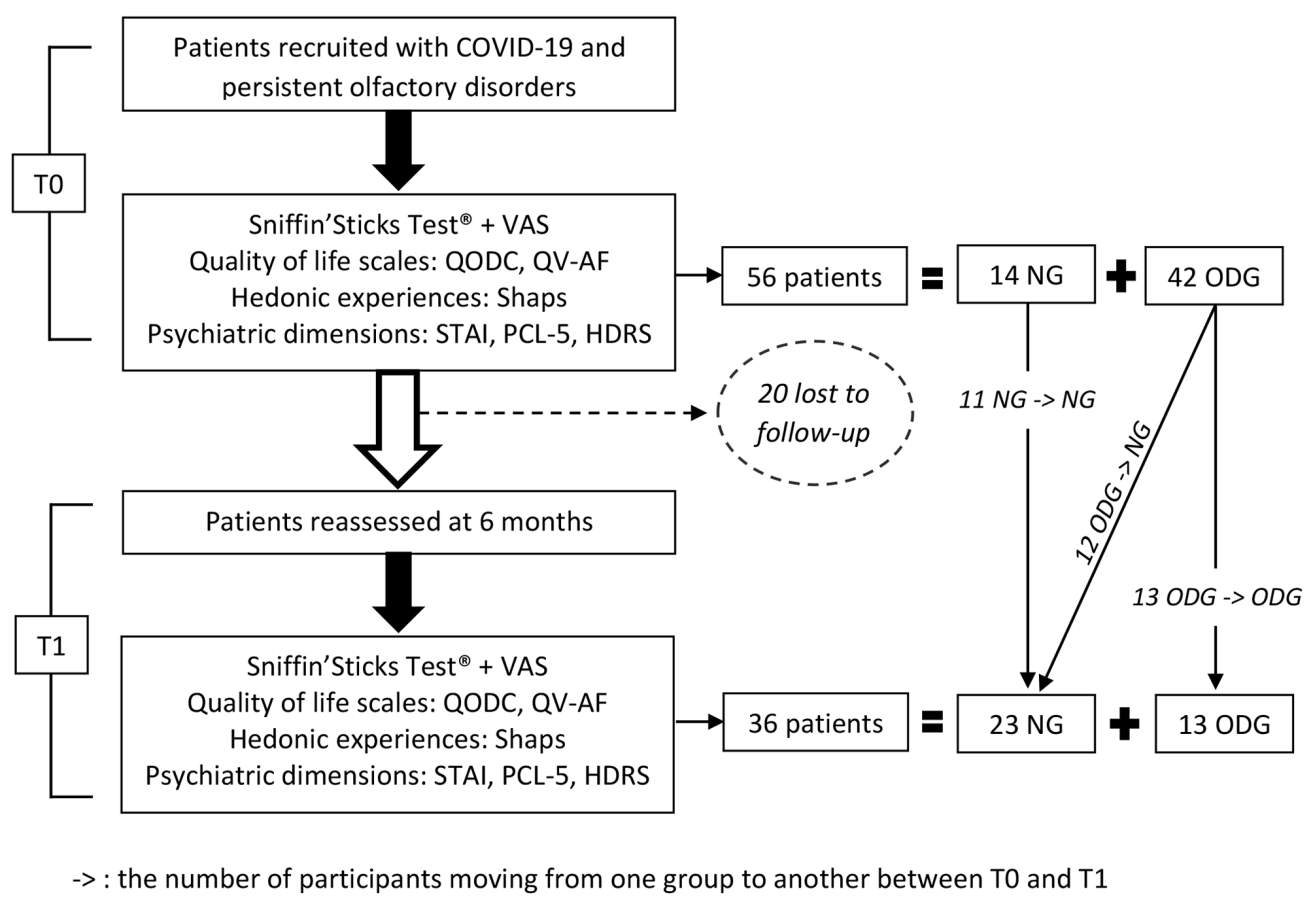
Flow chart

At T0, 56 patients were recruited, a majority of them being women, i.e. 33 (58.93%) vs. 23 men (41.07%). At T1, 36 patients were reassessed, 21 women (58.33%) and 15 men (41.66%). The average age of participants was 39.04 years (+/- 13.33). They presented post-COVID-19 olfactory disorders for an average of 5.32 (+/- 3.03) months. At T0, there were 14 patients in NG (TDI > 30.5) and 42 patients in ODG. At T1, there were 23 patients in NG and 13 patients in ODG. We lost to follow-up 20 patients between T0 and T1 (36% included at T0) (Fig. 1).

### Difference between the normosmic and olfactory disorder groups (Table 1)

At T0, there was no significant difference between two groups for quality of life, hedonic experiences, anxiety and depression dimensions. At T1, ODG had a significantly lower quality of life with QODC (*p* < 0.05) and QV-AF (*p* = 0.023) and more particularly in subscales “Food/pleasure” (*p* = 0.011) and “Food/psychology” (*p* = 0.012). ODG had also a significantly lower hedonic experience with Shaps than NG (*p* < 0.05). But there was no significant difference between the two groups regarding anxiety and depression dimensions.

**Table 1:** Descriptive analyses of the study populations with normosmic and with olfactory disorders

	T0				T1			
Variables	Mean (SD) *N* = 56	Normosmic (SD) *N* = 14	Olfactory disorder (SD) *N* = 42	*p*	Mean (SD) *N* = 36	Normosmic (SD) *N* = 23	Olfactory disorder (SD) *N* = 13	*p*
Age (years)	39.07 (13.33)	35.14 (11.35)	40.38 (13.80)	0.225				
Duration of olfactory disorder (month)	5.32 (3.03)	5.43 (2.77)	5.28 (3.15)	0.724				
TDI	24.73 (8.96)	36.11 (2.33)	20.93 (6.85)	< 0.05*	31.20 (10.49)	38.13 (3.51)	18.94 (6.59)	< 0.05*
Threshold (T)	4.83 (4.09)	10.32 (3.38)	3.01 (2.27)	< 0.05*	9.15 (5.24)	12.22 (3.57)	3.71 (2.57)	< 0.05*
Discrimination (D)	10.32 (3.05)	12.79 (1.31)	9.5 (3.02)	< 0.05*	11.39 (3.44)	13.22 (1.54)	8.15 (3.53)	< 0.05*
Identification (I)	9.57 (3.98)	13.00 (1.84)	8.42 (3.84)	< 0.05*	10.67 (3.52)	12.70 (1.96)	7.07 (2.69)	< 0.05*
VAS	35.39 (26.44)	47.86 (25.85)	31.24 (25.59)	0.031*	59.97 (32.02)	71.87 (22.94)	38.92 (35.68)	< 0.05*
QODC	11.21 (5.74)	12.71 (6.26)	10.71 (5.54)	0.267	13.14 (6.44)	15.96 (5.42)	8.15 (4.98)	< 0.05*
QVAF	253.60 (98.48)	281.21 (105.04)	244.40 (95.73)	0.194	271.94 (116.46)	301.50 (108.93)	219.62 (114.72)	0.023*
Food/pleasure	75.49 (23.13)	77.90 (18.81)	74.68 (24.55)	0.797	78.40 (21.87)	86.43 (13.73)	64.19 (26.59)	0.011*
Food/relational	60.43 (35.72)	68.94 (33.69)	57.59 (36.31)	0.327	62.96 (40.16)	70.30 (39.02)	49.97 (40.32)	0.121
Food/psychology	50.97 (32.58)	63.41 (34.23)	46.81 (31.33)	0.086	60.47 (39.68)	71.39 (36.82)	41.15 (38.39)	0.012*
Food/physical conditions	66.25 (34.49)	70.96 (31.66)	64.68 (35.60)	0.541	70.74 (36.59)	74.64 (34.58)	63.83 (40.40)	0.601
SHAPS	2.68 (2.23)	2.14 (2.35)	2.86 (2.19)	0.222	2.89 (2.60)	1.35 (1.75)	4.23 (2.89)	< 0.05*
STAI-AE	38.43 (12.84)	35.07 (14.41)	39.55 (12.25)	0.175	37.17 (14.12)	34.70 (13.67)	41.53 (14.36)	0.102
STAI-AT	42.20 (10.98)	38.71 (12.62)	43.36 (10.29)	0.127	42.42 (14.80)	40.26 (14.20)	46.23 (15.63)	0.269
PCL 5	15.36 (19.85)	13.36 (21.82)	16.02 (19.38)	0.509	13.64 (17.00)	10.57 (15.63)	19.07 (19.32)	0.171
HDRS	9.41 (7.41)	6.50 (5.00)	10.38 (7.87)	0.129	10.47 (7.05)	9.17 (6.92)	12.77 (6.94)	0.141

### Correlations between psychophysical tests and self-perceived olfactory evaluation of olfactory disorder (Table 2)

At T0, the psychophysical tests (TDI) and self-perceived olfactory evaluation (VAS) of ODG were significantly correlated with: quality of life (QODC) (rho = 0.367, *p* = 0.016 for TDI and rho = 0.369, *p* = 0.016 for VAS) and hedonic experiences (Shaps) (rho=-0.354, *p* = 0.021 for TDI and rho=-0.416, *p* = 0.006 for VAS).

At T1, the psychophysical tests was not significantly correlated with quality of life, hedonic experiences, anxiety and depression dimensions in the two groups of patients. The self-perceived olfactory evaluation of NG patient was significantly correlated with quality of life (QODC: rho = 0.520, *p* = 0.01 and QV-AF: rho = 0.546, *p* = 0.006), hedonic experience (Shaps: rho=-0.713, *p* = 0.0001), PTSD (PCL-5: rho=-0.534, *p* = 0.008) and depression (HDRS: rho=-0.544, *p* = 0.007). The self-perceived olfactory evaluation of ODG was only significantly correlated with the hedonic experience (Shaps: rho=-0.608, *p* = 0.027).

**Table 2:** Correlations of psychophysical tests (TDI) and self-perceived olfactory (VAS) olfactory evaluations

	T0							
	TDI Normosmic (*N* = 14)		TDI Olfactory disorder (*N* = 42)		VAS Normosmic (*N* = 14)		VAS Olfactory disorder (*N* = 42)	
	rho	p	rho	*p*	rho	*p*	rho	*p*
QODC	0.278	0.334	0.367	0.016*	0.467	0.091	0.369	0.016*
QVAF	0.219	0.451	0.122	0.439	0.253	0.381	0.178	0.258
*Food/pleasure*	− 0.031	0.915	0.250	0.109	0.121	0.677	0.215	0.170
*Food/relational*	0.244	0.399	0.097	0.537	0.264	0.360	0.165	0.296
*Food/psychology*	0.174	0.546	0.162	0.303	0.205	0.481	0.238	0.128
*Food/physical conditions*	0.412	0.142	-0.022	0.887	0.112	0.701	-0.028	0.855
SHAPS	0.029	0.92	-0.354	0.021*	− 0.068	0.817	-0.416	0.006*
STAI-AE	− 0.121	0.678	-0.084	0.594	− 0.090	0.758	-0.291	0.061
STAI-AT	0.019	0.946	-0.085	0.588	0.294	0.307	-0.020	0.898
PCL 5	0.141	0.628	0.251	0.107	− 0.157	0.589	0.147	0.35
HDRS	0.022	0.939	-0.013	0.931	0.026	0.927	-0.157	0.318
	T1							
	TDI Normosmic (*N* = 23)		TDI Olfactory disorder (*N* = 13)		VAS Normosmic (*N* = 23)		VAS Olfactory disorder (*N* = 13)	
	rho	*p*	rho	*p*	rho			
QODC	− 0.160	0.464	0.289	0.337	0.520	0.010*	0.163	0.593
QVAF	− 0.371	0.080	0.123	0.687	0.546	0.006*	0.082	0.788
*Food/pleasure*	− 0.076	0.730	-0.198	0.516	0.573	0.004*	-0.101	0.740
*Food/relational*	− 0.366	0.085	0.105	0.732	0.433	0.038*	0.162	0.595
*Food/psychology*	− 0.303	0.159	0.168	0.581	0.491	0.017*	-0.025	0.935
*Food/physical conditions*	− 0.293	0.174	0.018	0.951	0.338	0.114	-0.017	0.955
SHAPS	− 0.042	0.847	-0.318	0.289	− 0.713	0.0001*	-0.608	0.027*
STAI-AE	0.216	0.320	-0.046	0.879	− 0.329	0.124	0.132	0.667
STAI-AT	0.403	0.056	0.132	0.666	− 0.394	0.062	0.044	0.886
PCL 5	0.253	0.243	0.185	0.544	− 0.534	0.008*	0.185	0.544
HDRS	0.332	0.121	-0.187	0.539	− 0.544	0.007*	-0.377	0.202

## Discussion

This study aimed to assess the impact of post-COVID-19 olfactory disorders on the general population six months after infection. The findings revealed a significant difference between the group with olfactory disorders (ODG) and the normosmic group (NG) in terms of quality of life and hedonic experiences. However, no significant differences were observed in anxiety and depression dimensions.

In the NG, self-perceived olfactory evaluations were correlated with quality of life, hedonic experiences, and anxio-depressive dimensions. In the ODG, self-perceived olfactory evaluations were only correlated with hedonic experiences.

At both T0 and T1, there were significant differences between the NG and ODG in the TDI score (*p* < 0.05) and the VAS score (*p* = 0.04), affirming the appropriateness of the chosen tools for measuring olfactory disorders.

The significant differences observed between NG and ODG at 6 months in terms of quality of life and hedonic experiences align with findings in the literature [45, 46]. For instance, an online survey involving 322 COVID-19 positive individuals who had experienced a loss of smell or taste reported 87% reduced enjoyment of food, 56% decreased enjoyment of life in general, and 55% loss of appetite [18].

However, the study did not yield significant results regarding anxiety and depressive dimensions, which were not consistent with the initial hypothesis and the existing literature. This discrepancy could potentially be attributed to methodological factors. Schou et al. conducted a systematic review that included 66 studies (selected from a total of 1725 studies) and reported a higher prevalence of anxiety and/or depression (61%), fatigue (48%), cognitive deficits (41%), sleep disturbances (35%), and symptoms of post-traumatic stress disorder (30%) [17]. This systematic review also highlighted the methodological variability across these studies, including variations in study instruments and the timing of follow-up examinations [17]. In our study, it is possible to hypothesize that a reassessment of the patients after 6 months might have revealed significant results for the anxious and depressive dimensions, aligning with the literature [5, 6, 17, 18]. Although our results did not reach statistical significance, there was a notable trend towards worsening scores at 6 months in both groups, suggesting potential emerging issues in these dimensions.

The statistical comparison of the groups was carried out between the NG and ODG groups at T0 and T1. But it was difficult to compare the same group (NG or ODG) between T0 and T1, because between T0 and T1, the number of patients in each of these groups are different (from 14 patients at T0 to 23 patients at T1 in NG; from 42 patients at T0 in ODG to 13 patients at T1 in ODG). However, we can only observe a trend in the evolution of the results between T0 and T1 for the same group.In the ODG, all indicators have a statistical tendency to decreased at T1 except the T score (olfactory perception) and the VAS. The link between the T score and the EVA is consistent since it is the measurement of olfactory perception by psychophysical tests (TDI score) or by self-perceived olfactory evaluation (EVA). But the worsening of the TDI total score and scores D (olfactory discrimination) and I (olfactory identification) seems to follow the decrease in quality of life indicators. We can discuss idea that a lack of olfactory recovery (TDI score) promotes a deterioration in quality of life over time. We could add that the appreciation of olfactory quality seems to have more impact on quality of life than perception alone. In the NG, the hedonic experience (Shaps) and anxious dimension (STAI-AE) scores would have a tendency to decrease, possibly due to the long-Covid impact on psychological health of individuals, regardless of any involvement dealing with sensory impairments.

The results of our study highlight the impact of self-perceived olfactory (by VAS) on quality of life, hedonic experience, anxious and depressive dimensions, in subjects without psychophysical impairment (by TDI score).

These results suggest the influence of the subjectivity of a sensory perception that is even more important than the perceptual disorder itself. Lechien and al. also found difference between psychophysical and self-perceived olfactory impairment in their study: among the patients who had an olfactory complaint, only a third had an psychophysical disorder in olfactory evaluation [47]. The literature proposed some explanation of this results by compared specificities in post-COVID 19 olfactory disorder to a cold [6]. During a cold, the loss of smell appears during or immediately after having contracted it, it is easily attributed to the nasal congestion felt and is reversible when this congestion disappears [48]. In the case of COVID-19, the olfactory loss is not attributed to a perceived nasal blockage [47] but considered as a novel perception [6]. This sensory loss without causal attribution is so unusual that several patients in our study have precise memories of when they became aware of the loss of their sense of smell. Since this sudden olfactory loss is not attributable to a physical mechanism felt by the patient, its duration cannot be predicted between a few weeks to several months [7, 8]. These uncertainties could cause an emotional impact that contributes to olfactory subjectivity.

The emotional impact of self-perceived sensory impairment in the NG contributes to the risk of progression towards anxiety and depressive disorders [49]. The systematic review by Schou et al. reported that COVID-19 was a risk factor for PTSD [17]. Studies investigating olfactory impairment in populations with PTSD (veterans) [50] report increased sensitivity to odors reminiscent of trauma [51] and a disparity between self-perceived and psychophysical “sensitivity” to odors [50]. The literature supports too that reduced olfactory sensitivity accompanies depressive symptoms [52] and conversely, depressive symptomatology negatively impacts olfactory functioning [53], which could thus be taken as a marker of depression [54]. Thus the emotional impact would maintain the self-perceived olfactory impairment even if there is no longer any psychophysical olfactory disorder [55].

### Limitations

This study had several limitations that may have influenced the results. First of all, we note the small number of patients included in the study. This may have led to low statistical power of our results and explained some non-significant trends. We also retain a significant number of people lost to follow-up (36%) in this study. We can wonder about the reasons for leaving the study of patients who were not seen again at 6 months: was it a patient who had regained their sense of smell? Or on the contrary patients whose olfactory disorders would have worsened and/or too discouraged to continue the protocol? In any case, this may have had an impact on the results of the study. In addition, a significant number (*N* = 12) of ODG individuals at T0 became NG at T1: this may represent a selection bias and have an impact in the analysis of the results.

This study may also have confounding bias in unaccounted data. The medical and psychiatric histories of the patients included in the study were not taken into account in the protocol. We choose not to collected this data through the speech of the patients because we didn’t use a systematic way starting from a medical file standardized for the study. In the context of Post-COVID Condition Study, as defined by WHO, study participants could benefit from multidisciplinary care adapted to their symptoms. We selected patients in ENT specialist consultations with an olfactory complaint, which is a particularly significant symptom of Post-COVID condition and disruptive to patients’ quality of life. Other COVID-19-related symptoms that patients might have exhibited were not included in the data collection also due to the small sample size of the study. This may lead to a selection bias resulting in the difference between the two groups not being specifically linked to olfactory disorders.The data collected for the study made it possible to differentiate hyposmia, anosmia but also distorted olfaction. But due to the small size of the study sample and to keep group sizes statistically comparable, we grouped these symptoms in the ODG. This protocol is an ancillary study to research work focused on olfactory rehabilitation in post-COVID-19 olfactory disorders. During the evaluation period, the patients included in the study benefited from olfactory rehabilitation which they carried out alone at home, following the recommendations of the ENT specialist. The random observance of this rehabilitation on a small number of patients included and a large number of patients lost to follow-up did not make it possible to retain this variable as relevant. No correlation were found with quality of life, hedonic experience and anxious and depressive dimensions. Further studies with a larger number of patients are needed.

## Conclusion

This study has revealed a significant difference between the group with post-COVID-19 olfactory disorders and the normosmic group in terms of quality of life and hedonic experiences at 6 months. Moreover, the findings of this study suggest that the subjectivity of sensory perception plays a crucial role, which may be even more significant than the perceptual disorder itself. In line with existing literature, these results underscore the importance of a multidisciplinary clinical reassessment and psychological support as part of targeted sensory therapies.

It would be valuable to continue this research and explore the hypothesis that self-perceived olfactory evaluation, the subjective assessment of olfactory disorders, could act as a trigger for traumatic sensory experiences that may contribute to anxiety and depressive disorders. This avenue of investigation may lead to a deeper understanding of the psychological and sensory aspects of post-COVID-19 olfactory disorders and inform the development of more effective interventions and therapies.

## Data Availability

The reported data is part of an ongoing recording program. Anonymized participant data is not available for legal and ethical reasons. Anonymized data will be made available for research purposes upon reasonable request. Dr Louise-Emilie Dumas can be contacted in the event of a request for access to study data.

## Abbreviations

ENT specialist: Ear, nose and throat specialist
HDRS: Hamilton Rating Scale for Depression
NG: Normosmic group
ODG: Olfactory disorders group
PCL-5: Post Traumatic Stress Checklist Scale
PTSD: Post-Traumatic Stress Disorder
QOD: Questionnaire of Olfactory Disorders
QODC: Questionnaire of Olfactory Disorders– Short version
QV-AF: French quality of life and diet questionnaire
Shaps: Snaith-Hamilton Pleasure Scale
STAI: State-Trait Anxiety Inventory
TDI score: Threshold-Discrimination-Identification score
VAS: Visual Analog Scale
WHO: World Health Organization

## References

[1] Organisation Mondial de la Santé. Maladie à coronavirus 2019 (COVID-19): ce qu’il faut savoir [Internet]. 2021 [cited 2023 Mar 9]. Available from: https://www.who.int/fr/news-room/questions-and-answers/item/coronavirus-disease-covid-19.

[2] Brandão Neto D, Fornazieri M, Dib C, Di Francesco R, Doty R, Voegels R et al. Chemosensory Dysfunction in COVID-19: Prevalences, Recovery Rates, and Clinical Associations on a Large Brazilian Sample. Otolaryngol–Head Neck Surg Off J Am Acad Otolaryngol-Head Neck Surg [Internet]. 2021 Mar [cited 2023 Mar 9];164(3). Available from: https://pubmed.ncbi.nlm.nih.gov/32867582/. 10.1177/0194599820954825 PMC7464054 32867582

[3] Costa KVT da, Carnaúba ATL, Rocha KW, de Andrade KCL, Ferreira SMS, Menezes P. de L. Olfactory and taste disorders in COVID-19: a systematic review. Braz J Otorhinolaryngol [Internet]. 2020; Available from: https://www.ncbi.nlm.nih.gov/pmc/articles/PMC7280089/. 10.1016/j.bjorl.2020.05.008 PMC7280089 32580925

[4] Carrillo-Larco RM, Altez-Fernandez C. Anosmia and dysgeusia in COVID-19: A systematic review. Wellcome Open Res [Internet]. 2020;5. Available from: https://www.ncbi.nlm.nih.gov/pmc/articles/PMC7308993/. 10.12688/wellcomeopenres.15917.1 PMC7308993 32587902

[5] Moein S, Hashemian S, Tabarsi P, Doty R. Prevalence and reversibility of smell dysfunction measured psychophysically in a cohort of COVID-19 patients. Int Forum Allergy Rhinol [Internet]. 2020 Oct [cited 2023 Mar 9];10(10). Available from: https://pubmed.ncbi.nlm.nih.gov/32761796/. 10.1002/alr.22680 PMC7436559 32761796

[6] Doty RL (2022). Olfactory dysfunction in COVID-19: pathology and long-term implications for brain health. Trends Mol Med.

[7] Jafar A, Lasso A, Shorr R, Hutton B, Kilty S (2021). Olfactory recovery following infection with COVID-19: a systematic review. PLoS ONE.

[8] Boscolo-Rizzo P, Guida F, Polesel J, Marcuzzo AV, Antonucci P, Capriotti V (2022). Self-reported smell and taste recovery in coronavirus disease 2019 patients: a one-year prospective study. Eur Arch Oto-Rhino-Laryngol off J Eur Fed Oto-Rhino-Laryngol soc EUFOS Affil Ger soc Oto-Rhino-Laryngol. Head Neck Surg.

[9] Whitcroft KL, Hummel T (2020). Olfactory dysfunction in COVID-19: diagnosis and management. JAMA.

[10] Valsamidis K, Printza A, Constantinidis J, Triaridis S (2020). The impact of olfactory dysfunction on the psychological status and quality of life of patients with nasal obstruction and septal deviation. Int Arch Otorhinolaryngol.

[11] Ahmedy F, Mazlan M, Danaee M, Abu Bakar MZ (2020). Post-traumatic brain injury olfactory dysfunction: factors influencing quality of life. Eur Arch Otorhinolaryngol.

[12] Hosp J, Dressing A, Blazhenets G, Bormann T, Rau A, Schwabenland M et al. Cognitive impairment and altered cerebral glucose metabolism in the subacute stage of COVID-19. Brain J Neurol [Internet]. 2021 May 7 [cited 2023 Jul 27];144(4). Available from: https://pubmed-ncbi-nlm-nih-gov.proxy.unice.fr/33822001/. 10.1093/brain/awab009 PMC8083602 33822001

[13] Marin C, Vilas D, Langdon C, Alobid I, López-Chacón M, Haehner A (2018). Olfactory dysfunction in neurodegenerative diseases. Curr Allergy Asthma Rep.

[14] Schablitzky S, Pause BM. Sadness might isolate you in a non-smelling world: olfactory perception and depression. Frontiers in Psychology. Volume 5. Frontiers Media SA; 2014. 10.3389/fpsyg.2014.00045 PMC3916769 24570666

[15] Kohli P, Soler ZM, Nguyen SA, Muus JS, Schlosser RJ. The association between olfaction and depression: a systematic review. Chemical Senses. Volume 41. Oxford University Press; 2016. pp. 479–86. 10.1093/chemse/bjw061 PMC4918728 27170667

[16] Llana T, Méndez M, Garces-Arilla S, Hidalgo V, Mendez-Lopez M, Juan MC. Association between olfactory dysfunction and mood disturbances with objective and subjective cognitive deficits in long-COVID. Front Psychol [Internet]. 2023 Jan 16 [cited 2023 Jan 26];14. Available from: https://www.frontiersin.org/articles/10.3389/fpsyg.2023.1076743/abstract. 10.3389/fpsyg.2023.1076743 PMC9932904 36818111

[17] Schou TM, Joca S, Wegener G, Bay-Richter C (2021). Psychiatric and neuropsychiatric sequelae of COVID-19 - a systematic review. Brain Behav Immun.

[18] Coelho DH, Reiter ER, Budd SG, Shin Y, Kons ZA, Costanzo RM (2021). Quality of life and safety impact of COVID-19 associated smell and taste disturbances. Am J Otolaryngol.

[19] Tan HQM, Pendolino AL, Andrews PJ, Choi D. Prevalence of olfactory dysfunction and quality of life in hospitalised patients 1 year after SARS-CoV-2 infection: a cohort study. BMJ Open [Internet]. 2022;12(1). Available from: https://www.ncbi.nlm.nih.gov/pmc/articles/PMC8795927/. 10.1136/bmjopen-2021-054598 PMC8795927 35078845

[20] Liao B, Deng YK, Zeng M, Liu Z (2023). Long-term consequences of COVID-19: Chemosensory disorders. Curr Allergy Asthma Rep.

[21] Rass V, Tymoszuk P, Sahanic S, Heim B, Ausserhofer D, Lindner A (2023). Distinct smell and taste disorder phenotype of post-acute COVID-19 sequelae. Eur Arch Otorhinolaryngol.

[22] Hummel T, Liu DT, Müller CA, Stuck BA, Welge-Lüssen A, Hähner A (2023). Olfactory dysfunction: etiology, diagnosis, and treatment. Dtsch Ärztebl Int.

[23] Trecca EMC, Cassano M, Longo F, Petrone P, Miani C, Hummel T (2022). Results from psychophysical tests of smell and taste during the course of SARS-CoV-2 infection: a review. Acta Otorhinolaryngol Ital.

[24] Rumeau C, Nguyen DT, Jankowski R (2016). How to assess olfactory performance with the Sniffin’ sticks test®. Eur Ann Otorhinolaryngol Head Neck Dis.

[25] Hummel T, Sekinger B, Wolf SR, Pauli E, Kobal G (1997). Sniffin sticks’: olfactory performance assessed by the combined testing of odor identification, odor discrimination and olfactory threshold. Chem Senses.

[26] Hummel T, Konnerth C, Rosenheim K, Kobal G. Screening of olfactory function with a four-minute odor identification test: reliability, normative data, and investigations in patients with olfactory loss. Ann Otol Rhinol Laryngol [Internet]. 2001 Oct [cited 2023 Jan 26];110(10). Available from: https://pubmed.ncbi.nlm.nih.gov/11642433/. 10.1177/000348940111001015 11642433

[27] Zarachi A, Lianou A, Pezoulas V, Komnos I, Milionis O, Fotiadis D et al. Visual Analogue Scale for the Evaluation of Olfactory and Gustatory Dysfunction of COVID-19 Patients in Northwestern Greece. Cureus [Internet]. 2023 Mar 20 [cited 2023 Jul 27];15(3). Available from: https://pubmed-ncbi-nlm-nih-gov.proxy.unice.fr/37090302/. 10.7759/cureus.36413 PMC10115151 37090302

[28] De Sousa Machado A, Sousa F, Silva A, Meireles L. Visual Analog Scale and Olfactory Objective Tests in Hyposmia Patients: Is There a Link? Cureus [Internet]. 2023 Feb 7 [cited 2023 Jul 27];15(2). Available from: https://pubmed-ncbi-nlm-nih-gov.proxy.unice.fr/36909088/. 10.7759/cureus.34712 PMC9996390 36909088

[29] Heller G, Manuguerra M, Chow R. How to analyze the Visual Analogue Scale: Myths, truths and clinical relevance. Scand J Pain [Internet]. 2016 Oct [cited 2023 Jul 27];13. Available from: https://pubmed-ncbi-nlm-nih-gov.proxy.unice.fr/28850536/. 10.1016/j.sjpain.2016.06.012 28850536

[30] Brämerson A, Nordin S, Bende M. Clinical experience with patients with olfactory complaints, and their quality of life. Acta Otolaryngol (Stockh) [Internet]. 2007 Feb [cited 2021 Jul 12];127(2). Available from: https://pubmed.ncbi.nlm.nih.gov/17364348/. 10.1080/00016480600801357 17364348

[31] Mattos J, Edwards C, Schlosser R, Hyer M, Mace J, Smith T et al. A brief version of the questionnaire of olfactory disorders in patients with chronic rhinosinusitis. Int Forum Allergy Rhinol [Internet]. 2019 Oct [cited 2021 Jul 12];9(10). Available from: https://pubmed.ncbi.nlm.nih.gov/31430061/. 10.1002/alr.22392 PMC6773507 31430061

[32] Han P, Su T, Qin M, Chen H, Hummel T. A systematic review of olfactory related questionnaires and scales. Rhinology [Internet]. 2021 Apr 1 [cited 2023 Jan 26];59(2). Available from: https://pubmed.ncbi.nlm.nih.gov/33078172/. 10.4193/Rhin20.291 33078172

[33] Leclercq C, Chiesa-Estomba C, Horoi M, Le Bon S, Hans S, Distinguin L et al. Validity and Reliability of the French Short Version of the Questionnaire of Olfactory Disorders-Negative Statements (sQOD-NS). Ear Nose Throat J [Internet]. 2021 Aug 31 [cited 2023 Jan 26]; Available from: https://pubmed.ncbi.nlm.nih.gov/34463149/. 10.1177/01455613211032004 34463149

[34] Paineau D, Baudoin C, Grairia M, Rosset F, Bornet F, Zourabichvili O (2007). Développement Et validation d’une échelle de qualité de vie axée sur l’alimentation pour la population française. Cah Nutr Diététique.

[35] Snaith RP, Hamilton M, Morley S, Humayan A, Hargreaves D, Trigwell P (1995). A scale for the Assessment of Hedonic Tone the Snaith–Hamilton pleasure scale. Br J Psychiatry.

[36] Loas G, Dubal S, Perot P, Tirel F, Nowaczkowski P, Pierson A. Validation of the French version of the Snaith-Hamilton Pleasure Scale (SHAPS, Snaith et al. 1995). Determination of the statistical parameters in 208 normal subjects and 103 hospitalized patients presenting with depression or schizophrenia. L’Encéphale. 1997;23(6):454–8. 9488929

[37] Spielberger C, Gorsuch R, Lushene R, Vagg R (1983). Jacobs. Manual for the state-trait anxiety inventory: STAI.

[38] Gauthier J, Bouchard S (1993). Adaptation canadienne-française de la forme révisée du state–trait anxiety Inventory De Spielberger. Can J Behav Sci / Revue canadienne des Sci du Comportement.

[39] The PTSD Checklist (PCL): Reliability, validity, and diagnostic utility [Internet]. 1993 [cited 2022 Apr 16]; annual convention of the international society for traumatic stress studies…. Available from: https://scholar.google.com/citations?view_op=view_citation_hl=enuser=ddEs-1oAAAAJ_citation_for_view=ddEs-1oAAAAJ:u-x6o8ySG0sC.

[40] Yao SN, Cottraux J, Note I, De Mey-Guillard C, Mollard E, Ventureyra V. Evaluation des états de stress post-traumatique: validation d’une échelle, la PCLS. L’Encéphale. 2003 Mai-Juin;29(3 Pt 1):232–8. 12876547

[41] Blevins C, Weathers F, Davis M, Witte T, Domino J. The Posttraumatic Stress Disorder Checklist for DSM-5 (PCL-5): Development and Initial Psychometric Evaluation. J Trauma Stress [Internet]. 2015 Dec [cited 2022 Apr 16];28(6). Available from: https://pubmed.ncbi.nlm.nih.gov/26606250/. 10.1002/jts.22059 26606250

[42] Ashbaugh A, Houle-Johnson S, Herbert C, El-Hage W, Brunet A. Psychometric Validation of the English and French Versions of the Posttraumatic Stress Disorder Checklist for DSM-5 (PCL-5). PloS One [Internet]. 2016 Oct 10 [cited 2022 Apr 16];11(10). Available from: https://pubmed.ncbi.nlm.nih.gov/27723815/. 10.1371/journal.pone.0161645 PMC5056703 27723815

[43] Hamilton M. A rating scale for depression. J Neurol Neurosurg Psychiatry. 1960;23(1):56–62. 10.1136/jnnp.23.1.56 PMC495331 14399272

[44] Guelfi JD. L’évaluation clinique standardisée en psychiatrie. P. Fabre; 1996. 783 p.

[45] Otte MS, Haehner A, Bork ML, Klussmann JP, Luers JC, Hummel T. Impact of COVID-19-Mediated Olfactory Loss on Quality of Life. ORL J Oto-Rhino-Laryngol Its Relat Spec. 2023;85(1):1–6. 10.1159/000523893 PMC9148912 35413715

[46] Winter AL, Henecke S, Lundström JN, Thunell E. Impairment of quality of life due to COVID-19-induced long-term olfactory dysfunction. Front Psychol. 2023;14:1165911. 10.3389/fpsyg.2023.1165911 PMC10157159 37151341

[47] Lechien JR, Chiesa-Estomba CM, Hans S, Barillari MR, Jouffe L, Saussez S. Loss of Smell and Taste in 2013 European Patients With Mild to Moderate COVID-19. Ann Intern Med. 2020;173(8):672–5. 10.7326/M20-2428 PMC7505100 32449883

[48] Akerlund A, Bende M, Murphy C. Olfactory threshold and nasal mucosal changes in experimentally induced common cold. Acta Otolaryngol (Stockh). 1995;115(1):88–92. 10.3109/00016489509133353 7762392

[49] Vaira LA, Gessa C, Deiana G, Salzano G, Maglitto F, Lechien JR, et al. The Effects of Persistent Olfactory and Gustatory Dysfunctions on Quality of Life in Long-COVID-19 Patients. Life Basel Switz. 2022;12(2). 10.3390/life12020141 PMC8878431 35207429

[50] Cortese BM, Schumann AY, Howell AN, McConnell PA, Yang QX, Uhde TW. Preliminary evidence for differential olfactory and trigeminal processing in combat veterans with and without PTSD. NeuroImage Clin. 2017;17:378–87. 10.1016/j.nicl.2017.09.018 PMC5683811 29159050

[51] Wilkerson AK, Uhde TW, Leslie K, Freeman WC, LaRowe SD, Schumann A, et al. Paradoxical olfactory function in combat veterans: The role of PTSD and odor factors. Mil Psychol Off J Div Mil Psychol Am Psychol Assoc. 2018;30(2):120–30. 10.1080/08995605.2018.1425063 PMC6132269 30220788

[52] Pollatos O, Kopietz R, Linn J, Albrecht J, Sakar V, Anzinger A, et al. Emotional Stimulation Alters Olfactory Sensitivity and Odor Judgment. Chem Senses. 2007;32(6):583–9. 10.1093/chemse/bjm027 17495172

[53] Pabel LD, Hummel T, Weidner K, Croy I. The impact of severity, course and duration of depression on olfactory function. J Affect Disord. 2018;238:194–203. 10.1016/j.jad.2018.05.033 29886199

[54] Croy I, Symmank A, Schellong J, Hummel C, Gerber J, Joraschky P, et al. Olfaction as a marker for depression in humans. J Affect Disord. 2014 mai;160:80–6. 10.1016/j.jad.2013.12.026 24445134

[55] Bochicchio V, Mezzalira S, Maldonato NM, Cantone E, Scandurra C. Olfactory-related quality of life impacts psychological distress in people with COVID-19: The affective implications of olfactory dysfunctions. J Affect Disord. 2023;323:741–7. 10.1016/j.jad.2022.12.049 PMC9751003 36529409

